# Rare Disorder of Sexual Differentiation with a Mosaic 46,XX/47,XXY in a Klinefelter Syndrome Individual

**Published:** 2020

**Authors:** Preethi Pattamshetty, Harika Mantri, Vasavi Mohan

**Affiliations:** 1- Department of Genetics and Molecular Medicine, Vasavi Medical and Research Centre, Hyderabad, India; 2- Department of Genetics, Apollo Health and lifestyle Ltd, Hyderabad, India

**Keywords:** Counseling, Karyotyping, Klinefelter syndrome, Mosaicism, Sex determining region Y (SRY gene)

## Abstract

**Background:**

Klinefelter syndrome (KS) mosaicism 46,XX/47,XXY is an extremely rare disorder of sex development characterized by the presence of both ovarian and testicular tissues in the same individual. Both elements can be present in the same gonad (ovotestis) or separately in the same individual or as a unilateral ovotestis and the other side with testis or ovary. A mosaic with 46,XY would present with problems related to male infertility and in general, testicular insufficiency, but with a 46,XX mosaic, it is a completely rare presentation. As adolescents, these boys may experience severe emotional and behavioral issues; it is up to the parents to identify these conditions early and get them physician evaluated for possible abnormalities so that they can get the benefit of treatment.

**Case Presentation:**

A case of a rare disorder of sexual differentiation with a mosaic 46,XX/47,XXY in a KS individual is reported for whom karyotyping and SRY-FISH work-up was done.

**Conclusion:**

Early cytogenetic testing is essential to identify these individuals and testosterone replacement therapy and breast reduction for case management are helpful. Assisted reproductive technology (ART) may assist these individuals father children in some cases.

## Introduction

Sex chromosome aneuploidy as seen in Klinefelter syndrome is relatively common and affects 1:500 to 1:1000 male live births. This syndrome affects physical and to some extent, cognitive development, in males where there is at least one extra X chromosome added to the normal 46,XY male karyotype. It is characterized by hypogonadism, gynaecomastia, azoospermia/oligospermia, and increased levels of gonadotropins.

Mosaicism in Klinefelter syndrome has also been documented with 47,XXY/46,XY karyotype affecting about 10 % of cases. It is speculated that many of these cases arise due to paternal sperm disomy. On the other hand, 47 XXY/46 XX mosaicism with characteristics suggesting Klinefelter syndrome is very rare and to date, only seven or slightly more cases have been reported in the literature (1, 2). Meiotic non-disjunction of the sex chromosomes leads to altered dosage of sex chromosomes other than the normal XX or XY complement. Faculty of Medicine, Universiti Teknologi MARA, Sungai Buloh, Selangor Malaysia For men, the syndrome is picked up around puberty when the testes do not develop or when infertility issues arise as a couple tries for a pregnancy; early diagnosis can help in sex re-aligning and other management methods. Out of 1286 males referred to our center for infertility work-up or with hypergonadotropic hypogonadism or other behavioral issues, 17 males (1.3%) showed an XXY karyotype; one of them showed a rare mosaic with 47,XXY/46,XX chromosomes (0.08%).

## Case Presentation

A 15-year old person with male habitus presenting with complains of gynaecomastia was referred for evaluation in 2019, at Vasavi Medical and Research Centre, Hyderabad. The report showed a left undescended testicle (3×2.1 *cm*) (Cryptorchidism) with the evidence of a hypoechoic region of 1.1×0.9 *cm* in the left testis; there was probably ovarian tissue/neoplastic change and right testis was small, palpable in the scrotum and was operated for penoscrotal hypospadias.

Karyotyping report showed a mosaic with 33% 47,XXY cells and the remaining with an 46,XX karyotype.

### SRY gene testing by FISH:

Sex determining region Y (SRY gene) is also known as testis determining factor. This is a DNA binding protein that is a master regulator for male sex determination in humans. Individuals who have a normal Y chromosome and multiple copies of X chromosomes such as XXY are still males. While the presence or absence of SRY has generally determined whether or not testis development occurs, it has been suggested that there are other factors that affect the functionality of SRY.

In the present case, SRY/CCPX FISH Probe Kit was used to detect the human SRY gene located on chromosome band Yp11.31 along with the number of chromosome X copies per cell. LSP SRY FISH Probe covers a chromosomal region which includes the entire SRY gene. CCPX FISH Probe, derived from chromosome X-specific alpha satellite DNA, is designed to serve as a control to determine the number of chromosome X copies per cell but is also useful in detecting gene rearrangements involving the X chromosome. For cytogenetic analysis, briefly, 2 *ml* peripheral blood sample was collected from the patient in a sodium heparin vacutainer. Lymphocyte culture was done in RPMI 1640 medium to which peripheral blood and PHA was added and incubated at 37°*C* for 72 *hr*. Cell harvesting was done by adding colchicine and then the cells were treated with a hypotonic solution. After fixation using 3:1 methanol-acetic acid, G-banded metaphases were prepared for analysis and chromosomal study was performed. According to the guidelines of the International System for Human Cytogenetic Nomenclature (ISCN, 2016), karyotyping was done and numerical as well as structural abnormalities were recorded. Patients identified with chromosomal abnormalities were given post-test genetic counseling for appropriate management along with the referring clinician.

### Fluorescence in-situ hybridisation:

SRY gene detection was done using fluorescent DNA probes, which bind to specific regions of chromosomes in interphase (Non-dividing) cells. Peripheral blood received was centrifuged and cells were treated suitably and dropped onto slides. Probes were applied to blood cells. Next, 100 interphase cells for each probe were scored using a Leica microscope with Cytovision software.

## Discussion

Mosaic Klinefelter syndrome 46,XX is a rare entity. Association of lateral ovotesticular disorder with KS 46, XX/47, XXY requires an interdisciplinary approach for early diagnosis to resolve sex reassignment issues. The extra X chromosome may not entirely be inactivated and that is the reason why an increase in X chromosomes is associated with increased severity of presentation. A karyotype of 47,XXY may also be expected as mosaic with a 46,XY but the present case is rarely reported. Regular follow-up is necessary to prevent consequences arising due to gonadal insufficiency and inherent risk of malignancy. There have been studies reporting the low risk of malignancy in patients with the ovotesticular disorders. Pleskacova et al. (2010) have estimated the risk of malignancy to be 2.6% (3) whereas others reported the risk to be lying in between 2.6% and 4.6% (4). KS patients have increased risk of developing malignancy especially mediastinal germ cell tumors which makes follow-up in these patients essential. Song et al. (2014) have reported the risk of extra-gonadal germ cell tumors in young males with KS to be as high as 66.7% (5).

The presence of the SRY gene on the Y chromosome accounts for the male presentation of the individual. There have been cases of 47,XXY reported as females where there is SRY-negative Y chromosome, thus these individuals don’t identify as males. In the present case, though the karyotype showed only 33% of cells from peripheral blood to be 47,XXY cells, the individual essentially presented as a male. Klinefelter syndrome occurs mostly due to random genetic errors during the formation of egg or sperm after conception. It is congenital and some children experience other health comorbidities like autistic spectrum, increased height, low muscle tone, cardiovascular conditions, etc. The evaluation of the sexual phenotypic presentation and the gonadal phenotype can be rightly done with a complete work-up which includes karyotyping and SRY testing on the Y chromosome of these individuals which will ensure appropriate management options being delivered to the family.

## Conclusion

The boys with XXY/XX mosaicism may experience severe emotional and behavioral issues; it is up to the parents to identify these conditions early and get them physician evaluated for possible abnormalities so that they can get the benefit of treatment. As adolescents Testosterone replacement therapy and breast reduction can be done if early cytogenetic testing is done to identify these individuals. Assisted reproductive technology (ART) may help these individuals father children in some cases. Early intervention and testing is also important since many of these individuals may be at a risk of developing other disorders like type 2 diabetes, osteoporosis or blood vessels disorders. An increased risk of psychiatric disorders in these individual may also be attributable to the psychosocial pressures and lack of “fitting in” with their peer groups; appropriate family counseling can be given to help the individuals and reduce the risk for mental issues or cognitive impairment in these individuals.
